# Associations between social media exposure and COVID-19 preventive behaviors in post-zero-COVID China: a moderated mediation model of risk perception, fear, and mindfulness

**DOI:** 10.3389/fpubh.2026.1869399

**Published:** 2026-09-09

**Authors:** Ting Han, Miao Liu

**Affiliations:** 1 School of Digital Media and Design Arts, Beijing University of Posts and Telecommunications, Beijing, China; 2 School of Journalism and Communication, Beijing Normal University, Beijing, China

**Keywords:** COVID-19 preventive behaviors, fear, mindfulness, risk perception, social media exposure

## Abstract

**Background:**

In late 2022, the sudden termination of the “Zero-COVID” policy in China triggered a massive surge in infections and widespread public uncertainty. During this transition, social media served as the primary infrastructure for health communication. This study investigates the associations between social media and COVID-19 preventive behaviors, specifically examining the mediating roles of risk perception and fear, and the moderating influence of mindfulness.

**Objective:**

This study aims to investigate the psychological pathways linking social media exposure to COVID-19 preventive behaviors during the abrupt termination of China’s “Zero-COVID” policy in late 2022. Specifically, the research examines the parallel mediating roles of cognitive risk perception and fear, while evaluating the potential of mindfulness as a self-regulatory resource to moderate the internal states and preventive health behaviors during a public health crisis.

**Methods:**

A cross-sectional online survey was conducted among 1,167 Chinese adults between December 21, 2022, and January 3, 2023—the peak period of the policy shift. Data were analyzed using the SPSS PROCESS macro. Mediation effects (Model 4) were tested for risk perception and fear, while a moderated mediation model (Model 14) was employed to evaluate whether mindfulness buffered the relationship between fear and preventive action.

**Results:**

This study found that social media exposure was positively associated with risk perception (*β* = 0.600, SE = 0.142, *p* < 0.001), which in turn, was positively associated with preventive behaviors (*β* = 0.069, SE = 0.012, *p* < 0.001). As revealed by the proposed model, social media exposure was positively associated with fear of COVID-19 (*β* = 0.145, SE = 0.034, *p* < 0.001). Fear of COVID-19 was positively associated with preventive behaviors (*β* = 0.238, SE = 0.047, *p* < 0.001), social media exposure showed a significant overall indirect association with preventive behaviors through fear (*β* = 0.034, 95% CI = 0.013, 0.062). Besides, mindfulness moderated the association between fear and COVID-19 preventive behaviors (*β* = 0.170, SE = 0.046, *p* < 0.001). The indirect effect of social media exposure on COVID-19 preventive behaviors through fear was significant for mindfulness at the moderate (effect = 0.024, SE = 0.010, BootLLCI = 0.006, BootULCI = 0.047), and high (effect = 0.043, SE = 0.015, BootLLCI = 0.017, BootULCI = 0.075) levels.

**Conclusion:**

Social media exposure is indirectly associated with preventive actions through risk perception and fear. Notably, mindfulness serves as a critical boundary condition: the positive link between threat-induced fear and adaptive protective behaviors is statistically significant only for individuals with moderate-to-high mindfulness. This study highlights the importance of incorporating emotional regulation into public health communication. Rather than relying solely on fear-inducing information, future communication strategies should couple risk information with digital mindfulness resources to support public health compliance and mental well-being during post-policy transitions.

## 1 Introduction

In late 2022, China underwent a historic transition in its pandemic management strategy. Following nearly 3 years of stringent “Zero-COVID” policies, the government issued the “Notice on Further Optimizing the Implementation of the Prevention and Control Measures of the COVID-19 Epidemic” (1). This abrupt transition triggered an unprecedented surge in infections and created a unique “information vacuum,” as the public was forced to shift from state-mandated protection to self-regulated preventive behaviors almost overnight. In this state of collective psychological flux, social media emerged as the primary infrastructure for health communication, filling the gap left by rapidly evolving official guidelines.

While the link between media exposure and behavioral intent is well-documented, the mechanism underlying how psychological responses translate into adaptive behaviors is not yet clear. Specifically, it remains unclear why the link between fear and preventive behaviors varies significantly across individuals. This study proposes that mindfulness, which is the capacity to maintain non-judgmental, present-moment awareness, serves as a vital self-regulatory resource. By positioning mindfulness as a cognitive and emotional buffer, we can better understand how individuals maintain behavioral resilience despite the stress of a public health crisis. Previous studies have primarily examined the associations between social media exposure and preventive behaviors during the early stages of the pandemic, when preventive behaviors were largely shaped by institutional mandates and public health policies. However, the post–Zero-COVID transition represented a shift toward greater individual responsibility for health risk assessment and preventive decision-making. This context provides a unique opportunity to examine how individuals interpret rapidly changing digital health information, develop risk perceptions and emotional responses, and translate these psychological processes into preventive actions. Existing studies have largely examined whether social media exposure predicts preventive behaviors. This study advances this literature by examining under what psychological conditions threat-related responses are associated with preventive actions during a major public health transition.

Building on the threat appraisal perspective of the Extended Parallel Process Model (EPPM), this research investigates the psychological mechanisms associated with social media exposure and COVID-19 preventive behaviors during China’s post–Zero-COVID transition. Rather than testing the complete EPPM framework, which emphasizes efficacy beliefs, this study focuses on how threat-related cognitive and emotional responses are associated with preventive actions in a changing information environment. Furthermore, drawing on self-regulation perspectives, this study investigates whether mindfulness moderates the association between fear and preventive behaviors by considering individual differences in the regulation of threat-related emotions. Taken together, this study aims to provide a more nuanced understanding of the underlying mechanisms linking social media exposure to preventive behaviors in digitally mediated environments. Therefore, this study extends previous public health research by demonstrating how threat-related cognitive and emotional responses are associated with preventive behaviors in a digitally mediated health environment, where individuals increasingly rely on online information and personal risk assessment to guide health decisions. The findings aim to provide evidence-based insights for health authorities to design communication strategies that promote sustainable protective behaviors without compromising public mental health.

### 1.1 Social media exposure, perceived risk, and COVID-19 preventive Behaviors

Social media has become a powerful platform for the rapid dissemination of health information and the promotion of healthy behaviors, particularly in times of crisis (2). Thanks to the immediacy, accessibility, and interactivity of social media, people can promptly access health-related information and participate in preventive actions (3–5). During the COVID-19 pandemic, encouraging individuals to actively engage in preventive actions was one of the core themes of COVID-19 in social media (6). Therefore, it is imperative to investigate the link between social media exposure and the COVID-19-related preventive behaviors.

Beyond information dissemination, social media also plays a crucial role in shaping individuals’ psychological responses. As a primary channel for people to access health information (5, 7–10), social media play an important role in affecting the psychological and behavioral responses during the COVID-19 pandemic. Besides, social media have been widely identified to be significantly associated with people’s risk perception (11–13). Risk perception refers to people’s intuitive assessment of the danger to which they are or may be exposed (14), including their subjective judgment of the risk characteristics, probability, and severity (15). According to The Social Amplification of Risk Framework, the media can act as a social amplifier to either amplify or weaken people’s risk perception through agenda setting (16). The linkage between social media and risk perception has been verified in studies on avian influenza (17), H1N1 (18), and MERS (13). In risk setting, social media can intensify the flow and diffusion of risk information to a certain extent, thus enhancing risk perception in individuals (19–21). Moreover, risk perception has been extensively indicated to be positively associated with the preventive health behaviors (12, 22). As stated in the Extended Parallel Process Model, individuals who perceive no risk will ignore the problem and take no action (23). When people perceive a higher risk, they can actively engage in preventive health behaviors to protect their health (24–27). Therefore, the following hypotheses were proposed:

*H1*: Social media exposure is positively associated with risk perception.

*H2*: Risk perception mediates the association between social media exposure and preventive behaviors related to COVID-19.

### 1.2 Social media exposure, fear of COVID-19, and preventive Behaviors

In addition to cognitive evaluations, social media exposure is also linked to emotional responses. Although social media can generate certain positive emotional responses including hope among users (28), a growing body of research highlights its adverse emotional impacts, particularly fear-inducing effects (29), during the COVID-19 pandemic (30). During public health crises, social media platforms often amplify emotionally charged content, including alarming statistics, personal narratives, and misinformation (31, 32). Fear is a strong and distressing emotion that is activated in response to a perceived threat (33–38). Public health crisis formation on social media is usually constructed emotionally (39). Research has shown that, negative emotions (such as fear) are more likely to be disseminated on social media than positive emotions (40–43). During the COVID-19 pandemic, widespread exposure to fear-inducing content—such as reports of fatalities, overwhelmed healthcare systems, and misinformation—contributed to heightened public anxiety (44, 45). Many previous studies have shown that social media exposure is positively associated with fear such that people who use social media more frequently are more likely to develop a fear of COVID-19 (44, 46–51). Recent research on COVID-19 has also pointed out problematic social media use (52, 53) and Social Network Use Disorder (54) were related to fear of COVID-19.

From a behavioral perspective, fear is recognized as a pivotal correlate of adaptive health behaviors. Furthermore, social media may also be linked to preventive behaviors through heightened fear of COVID-19. Fear is a driving force that motivates people to adopt health behaviors (55, 56). Previous studies have shown the association between fear of COVID-19 and various health behaviors. For example, fear of COVID-19 has been found to promote the purchase of protective equipment (57), vaccination (58–61), preventive behaviors related to COVID-19 (62, 63), and reduce travel intentions (64, 65). Moreover, empirical evidence suggests that fear represents an important psychological process associated with the relationship between external stimuli and preventive actions. Ahorsu et al. (66) found that fear of COVID-19 significantly mediates the association between perceived health status and insomnia, mental health, and preventive behaviors related to COVID-19. Serpas and Ignacio (67) reported that fear of COVID-19 fully mediated the association between risk perception and COVID-19 preventive behaviors. Building on previous research, we argue that social media exposure may indirectly be linked to preventive behaviors by eliciting fear.

*H3*: Social media exposure is positively associated with fear of COVID-19.

*H4*: Fear of COVID-19 mediates the association between social media exposure and preventive behaviors related to COVID-19.

### 1.3 The moderating role of mindfulness

While both risk perception and fear can motivate preventive behaviors, individuals differ in how they regulate these psychological responses. This study proposes mindfulness as a key moderating factor that shapes the translation of internal states into behavioral outcomes. Mindfulness refers to the capacity to focus full attention on the process and content (e.g., physical sensations, emotions, imaginations, states of mind, appearances, etc.) of a mental activity taking place in the present moment without judgment or acceptance (68–73). More precisely, mindfulness emphasizes the feeling of being fully present and alive in the moment (69, 74). The individual needs to bring a certain quality of attention to the moment-to-moment experience (75), and to accept the experience of the present moment with non-judgment, openness, and identification (76, 77). In this mental process, if we can focus on and accept our present thoughts, emotions, and actions without judgment, then the experiences, whether good or bad, will not affect us (78). As a protective factor, mindfulness is an essential psychological buffering mechanism and self-regulation strategy (69, 79, 80), and has been adopted as an approach to increasing awareness and responding skillfully to mental processes that contribute to emotional distress and maladaptive behavior (74). Mindfulness not only can effectively alleviate negative emotions such as anxiety (81, 82), depression (83), stress (84, 85), and job burnout (78, 86, 87), but also promote better diet (88), more vaccinations (89), more physical activity (90), safer sexual behavior (90), smoking cessation (91), and other health behaviors. Therefore, mindfulness is considered a valuable personal resource for regulating mental health and improving health behaviors (69, 85, 92, 93). It is necessary to investigate the regulatory function of mindfulness in the context of the COVID-19 pandemic.

While the link between social media and behavioral intent is documented, the psychological boundary conditions—specifically, why some individuals successfully translate fear into action while others succumb to distress—remain under-explored. This study fills this gap by positioning mindfulness as a cognitive buffer that regulates the transition from distress to proactive health behavior. So far, only a few studies have examined the role of mindfulness in the context of COVID-19 (93–96). These studies reported associations between mindfulness, fear of COVID-19, and risk perception. It has been found that higher levels of mindfulness correlate with less COVID-19 fear (93, 94, 96) and risk perception (95). As a crucial self-regulatory capacity, mindfulness may serve as a buffer that moderates the associations between risk perception, fear of COVID-19, and their subsequent behavioral correlates.

To model these complex dynamics, this study draws upon the conceptual strengths of the Extended Parallel Process Model (EPPM) and Self-Regulation Theory. The EPPM stands as one of the representative frameworks for predicting individuals’ health behaviors, enabling a more nuanced understanding of the mechanisms shaping COVID-19 preventive behaviors. The EPPM proposes that fear appeals influence behavior through individuals’ evaluations of perceived threat and perceived efficacy (97). When people perceive a serious and personally relevant threat and believe they can effectively respond to it, they engage in danger control (protective action); otherwise, they may engage in fear control (defensive avoidance or denial) (23). The sudden transition from state-led protection to self-directed health management required substantial cognitive and emotional resources from individuals. Under conditions of limited healthcare availability and social support, the role of self-efficacy may have become more context-sensitive, motivating the present study to focus specifically on the threat appraisal component of the EPPM framework. This approach is particularly relevant in China’s post–Zero-COVID transition, which was characterized by heightened informational uncertainty and increased individual responsibility for health protection. During this period, individuals increasingly relied on personal judgments to assess infection risk and develop appropriate preventive actions. Therefore, rather than attempting to test the complete EPPM framework, this study examines how cognitive evaluations of risk perception and affective responses of fear jointly relate to COVID-19 preventive behaviors.

**Figure 1:**
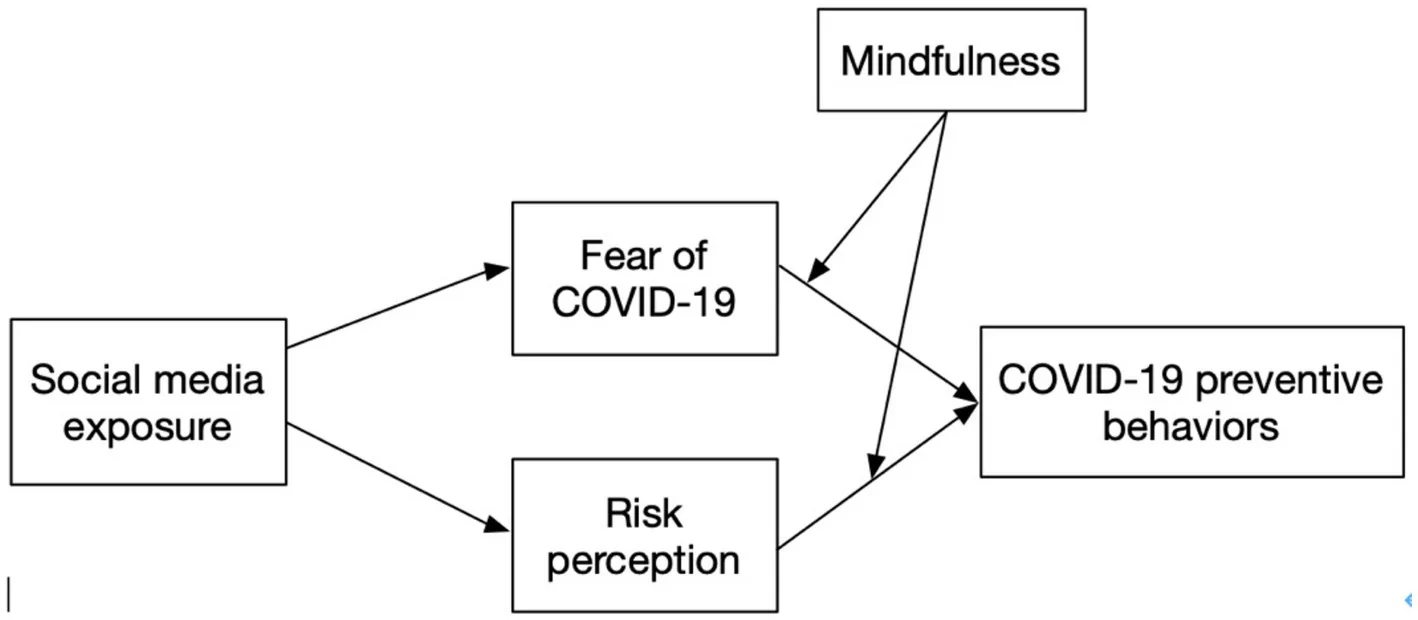
Conceptual framework.

However, fear does not always exhibit a consistent relationship with adaptive behaviors, as individuals may differ in their regulatory resources to cope with emotional responses. Building on self-regulation theory, mindfulness may serve as a regulatory resource that helps individuals manage fear-related arousal and maintain attention toward health-protective goals. Self-regulation theory explains the cognitive and behavioral mechanisms through which individuals track, evaluate, and modify their own conduct in pursuit of specific targets (98). Within this framework, mindfulness functions as a vital internal resource that enhances awareness and attentional control. Consequently, it enables individuals to objectively evaluate situational risks and regulate negative emotional responses without losing sight of their health-protective goals. Accordingly, this study proposes a moderated mediation model (see Figure 1) in which social media exposure is associated with COVID-19 preventive behaviors through two parallel pathways: cognitive appraisal (risk perception) and affective response (fear). Furthermore, mindfulness is examined as an individual-level regulatory capacity that may shape the extent to which threat-related cognitions and emotions are translated into adaptive protective actions.

## 2 Method

All participants were randomly recruited from databnu.com, a national online panel comprising 1,075,809 Chinese adults. Participants in the online panel of databnu.com were recruited through both online and offline channels. Mini programs embedded in social media platforms such as WeChat were used to recruit participants online, and research representatives were used to recruit participants offline. Data were collected using Qualtrics, a web-based survey software, from December 21, 2022, to January 3, 2023. Data collection was conducted after the termination of the strict “ZERO-COVID” policy in early December 2022, a period during which nearly 250 million people in China might have contracted COVID-19. The study was approved by the Institutional Review Board (IRB) of Beijing Normal University. All participants signed an online informed consent form before completing the questionnaires. After the web-based survey was completed, we conducted data cleaning procedures using Excel. First, we removed duplicate cases, including those with duplicate IP addresses and duplicate entries. Then, participants who did not complete the survey or completed it in less than 7 min were excluded from the final analysis. After data cleaning procedures, a total of 1,167 respondents were retained. Table 1 presents the descriptive statistics for the samples.

**Table 1:** Respondents’ characteristics (*N* = 1,167).

Demographics	*N*	%
Gender		
Female	739	63.4%
Age		
<18	33	2.8%
18–34 years	819	70.3%
35–44 years	243	20.9%
45–54 years	54	4.6%
>55 years	16	1.4%
Education		
Primary or secondary school	120	10.2%
High school	244	20.9%
Associate degree	343	29.4%
College	439	36.9%
Graduate degree	28	2.4%
Annual family income		
≦50,000 rmb	294	25.2%
50,000–100,000 rmb	415	35.6%
100,000–200,000 rmb	341	29.3%
200,000–500,000 rmb	84	7.2%
>500,000 rmb	31	2.7%

### 2.1 Measurement

#### 2.1.1 Social media exposure

In this study, social media exposure was conceptualized as the frequency of encountering COVID-19-related information across major platforms rather than general social media use. Respondents were asked to indicate the frequency with which they encountered COVID-19-related information on four major social media platforms—WeChat, Weibo, Douyin (TikTok), and Kuaishou—using a 5-point scale ranging from 1 (never) to 5 (very frequently) (M = 3.39, SD = 0.75). The measure captured the overall frequency of exposure to COVID-19-related information and did not distinguish among specific content characteristics, such as information valence, source, or evidentiary basis. We created an additive index by combining the four questions.

#### 2.1.2 Fear

Fear was measured by the fear of COVID-19 Scale developed by Ahorsu, Lin, Imani, et al. (99). Fear of COVID-19 was assessed based on a Likert scale that was scored from one to seven. Sample items include: “I have avoided using public transport because of the fear of contracting coronavirus (COVID-19),” “I cannot sleep because I’m worrying about getting coronavirus-19,” and “My hands become clammy when I think about coronavirus-19.” The internal reliability (Cronbach’s alpha) is 0.89 (*M* = 2.68, *SD* = 0.87).

#### 2.1.3 Risk perception

Risk perception was measured by a 5-item Likert scale developed by Siegrist and Bearth (100). The participants answered on a scale from 1 (no fear) to 7 (very high fear). Sample items include: “Related to the new coronavirus, I am afraid that I will be infected,” “I am afraid that someone from my family or my acquaintances will be infected,” and “I am afraid that there will be fatalities in my social environment.” The internal reliability (Cronbach’s alpha) is 0.92 (M = 3.50, SD = 1.73).

#### 2.1.4 Preventive behavior

Preventive behavior was measured by a 5-item Likert scale developed by Kamran et al. (101), which the participants answered on a scale from 1 (never) to 5 (very often). Sample items include: “Wearing gloves outside the home” and “Minimize public presence such as purchases and banking.” The internal reliability (Cronbach’s alpha) is 0.78 (*M* = 3.98, *SD* = 1.37).

#### 2.1.5 Mindfulness

Mindfulness was assessed using the Mindful Attention Awareness Scale (MAAS) (69). The scale includes 6 items, such as “I was finding it difficult to stay focused on what was happening” and “I was rushing through something without being really attentive to it.” Participants are instructed to indicate their degree of agreement on each item, ranging from 1 (not at all) to 6 (very much). The mindfulness scale exhibited satisfactory internal reliability (Cronbach’s α = 0.91) (*M* = 2.27, *SD* = 0.90).

#### 2.1.6 Demographic variables

Age, gender, family annual income in Chinese Yuan from 1 = CNY 50000 or below to 5 = CNY 500000 or higher, and education (ranging from 1 = Primary or secondary school to 5 = Graduate degree) were measured as demographic variables.

The construct validity of the measurement models was assessed using confirmatory factor analysis (CFA). The results demonstrated good fit to the data, supporting the validity of the study measures. Internal consistency was evaluated using Cronbach’s alpha coefficients, and all scales demonstrated satisfactory reliability.

### 2.2 Data analysis

Pearson correlation analysis was conducted to evaluate the correlation between the analyzed variables. To test the mediating roles of fear of COVID-19 and risk perception, a bootstrapping analysis utilizing PROCESS MODEL 4 was employed. Process MODEL 4 is used for simple mediation and allows for testing multiple mediators simultaneously. To test whether the indirect path (i.e., social media use → fear →COVID-19 preventive behaviors) was contingent upon mindfulness, PROCESS MODEL 14 was employed for analyzing the proposed model. MODEL 14 is used for moderated mediation and allows to test the mediation effect and the moderation effect simultaneously. The proposed model is illustrated in Figure 1.

## 3 Results

**Figure 2:**
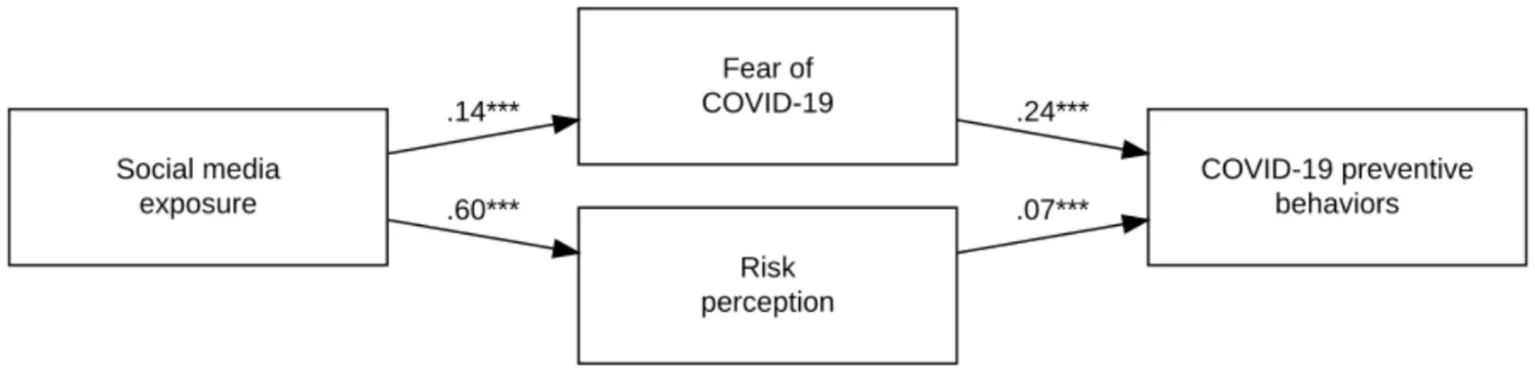
The results of mediation analysis. Unstandardized path coefficients are reported. ***p < 0.001.

**Figure 3:**
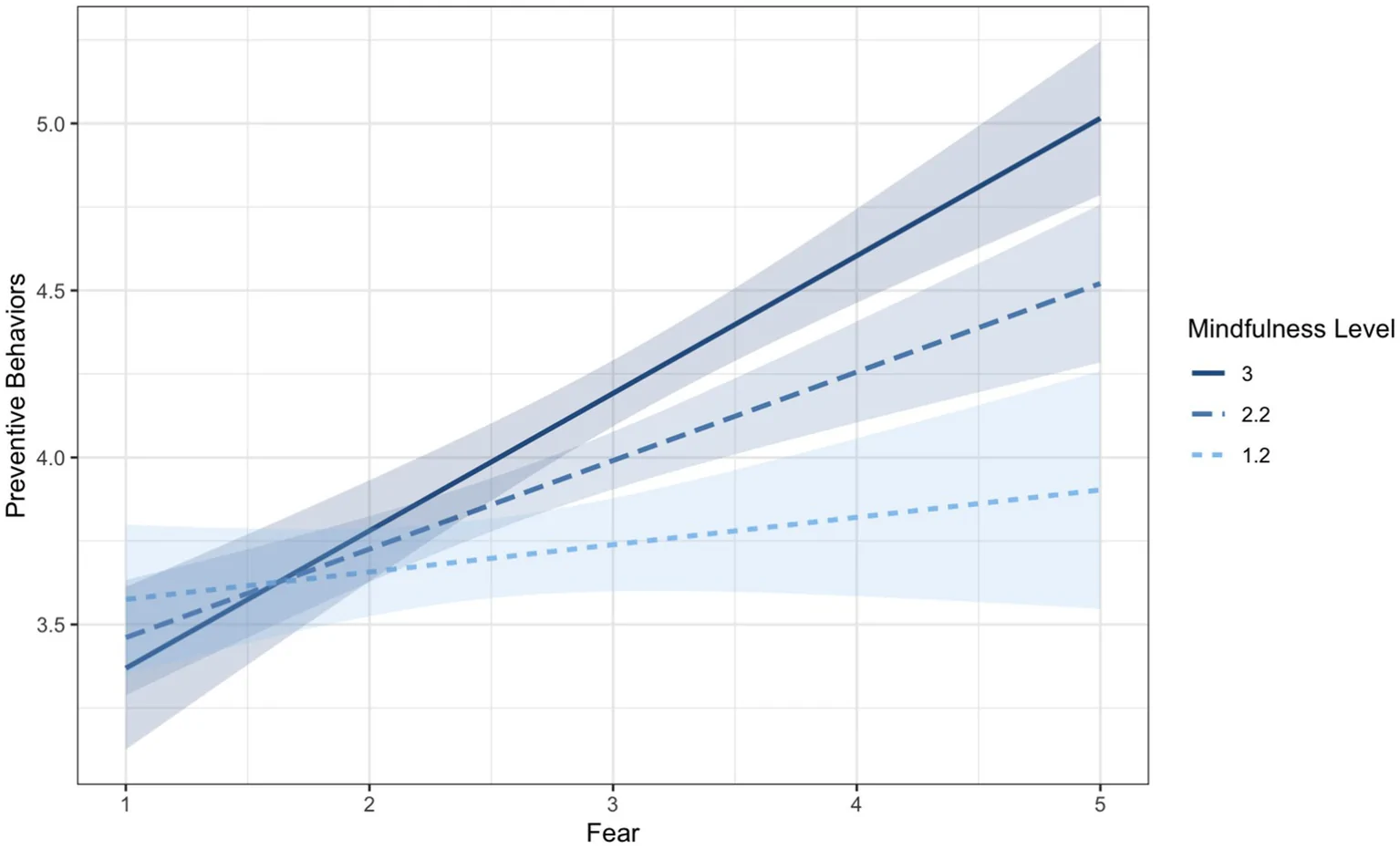
Simple slope plot of the interaction between fear of COVID-19 and mindfulness on preventive behaviors.

Before testing the conceptual model, we evaluated the potential for common method bias (CMB) since all data were self-reported. Harman’s single-factor test was conducted by including all items of the key constructs in a principal component analysis. The results showed that the first factor accounted for 35.58% of the variance, which is below the 40% threshold, indicating that CMB was unlikely to substantially influence the findings (102). To assess the mediating role of fear of COVID-19 and risk perception in the relationship between social media exposure and COVID-19 preventive behaviors, PROCESS Model 4 was employed to test H1 and H2, with age, gender, and health knowledge being the covariates. It was found that social media exposure was positively associated with risk perception (*β* = 0.600, SE = 0.142, *p* < 0.001), which in turn, was positively associated with preventive behaviors (*β* = 0.069, SE = 0.012, *p* < 0.001), supporting H1 and H2. The linkage between social media exposure and preventive behavior was significant (*β* = 0.259, SE = 0.051, *p* < 0.001). As revealed by the proposed model, social media exposure was positively associated with fear of COVID-19 (*β* = 0.145, SE = 0.034, *p* < 0.001), which supported H3. Fear of COVID-19 was positively linked to preventive behaviors (*β* = 0.238, SE = 0.047, *p* < 0.001). Furthermore, a significant indirect linkage was observed between social media exposure and preventive behaviors, mediated by fear (*β* = 0.034, SE = 0.013, BootLLCI = 0.013, BootULCI = 0.062), supporting H4. The results of the mediating analysis are shown in Figure 2. To further examine the moderated mediation effect, PROCESS model 14 was used. The conditional indirect effect of social media exposure on preventive behaviors through fear of COVID-19 was assessed at three levels of mindfulness (i.e., Mean - 1SD, Mean, and Mean + 1SD). After introducing mindfulness as a moderator, the direct associations of fear and risk perception with preventive behaviors were no longer statistically significant. Instead, the interaction term between fear and mindfulness was statistically significant (*β* = 0.170, SE = 0.046, *p* < 0.001), indicating that the association between fear and preventive behaviors varied across different levels of mindfulness. The indirect association between social media exposure and COVID-19 preventive behaviors via fear was significant at moderate level (effect = 0.024, SE = 0.010, BootLLCI = 0.006, BootULCI = 0.047) and high level (effect = 0.043, SE = 0.015, BootLLCI = 0.017, BootULCI = 0.075) of mindfulness, as the confidence intervals did not contain zero. However, the indirect effect of social media exposure on preventive behavior via COVID-19 fear was not significant for mindfulness at the low level (effect = −0.001, SE = 0.013, BootLLCI = −0.027, BootULCI = 0.024). Therefore, fear of COVID-19 exhibited a significant positive association with preventive behaviors at two levels of mindfulness. For individuals who had a lower level of mindfulness, the relationship between fear and preventive behaviors was not significant. Figure 3 presents the simple slope plot of the interaction between fear of COVID-19 and mindfulness on preventive behaviors. We also found that mindfulness was not a significant moderator for the relationship between risk perception and preventive behaviors (*β* = 0.012, SE = 0.013, BootLLCI = −0.014, BootULCI = 0.038). The indirect effect of social media exposure on preventive behavior via perceived risk is not significant for mindfulness at low, medium and high level (Index = 0.007, Boot SE = 0.011, BootLLCI = −0.010, BootULCI = 0.032). The results revealed that the index of Moderated Mediation for COVID-19 fear was statistically significant (Index = 0.025, Boot SE = 0.011, BootLLCI = 0.007, BootULCI = 0.047). The Index of Moderated Mediation for the risk perception pathway was not statistically significant. Compared with the mediation model, the moderated mediation model increased *R*^2^ from 0.150 to 0.175. Table 2 shows the results of the mediation model and the moderated mediation model.

**Table 2:** The result of the mediation model and the moderated mediation model.

COVID-19 preventive behaviors	Coefficient	SE	*t*	*p* value	LLCI	ULCI
Mediation model						
Social Media Exposure (X)	0.259	0.051	5.098	<0.001	0.159	0.358
Fear (M1)	0.238	0.047	5.015	<0.001	0.145	0.331
Risk Perception (M2)	0.069	0.012	5.978	<0.001	0.046	0.091
*R^2^*	0.1500					
Indirect effect (X → M1 → Y)	0.034	0.013			0.013	0.062
Indirect effect (X → M2 → Y)	0.041	0.013			0.019	0.069
Indirect effect	0.076	0.019			0.041	0.116
Moderated mediation model						
Social Media Exposure (X)	0.237	0.050	4.724	<0.001	0.139	0.336
Fear (M1)	−0.212	0.117	−1.814	0.070	−0.441	0.017
Risk Perception (M2)	0.038	0.028	1.371	0.171	−0.017	0.093
Mindfulness (W)	−0.446	0.135	−3.311	0.001	−0.711	−0.182
M1 * W	0.170	0.046	3.686	<0.001	0.080	0.261
M2 * W	0.012	0.013	0.927	0.354	−0.014	0.038
*R^2^*	0.1751					
Index of moderated mediation (M1)	0.025	0.011			0.007	0.047
Index of moderated mediation (M2)	0.007	0.011			−0.010	0.032

SE, standard error, LLCI, lower level of confidence interval, ULCI, upper level of confidence interval.

## 4 Discussion

The present study is designed to timely assess how Chinese people engaged in COVID-19 preventive behaviors right after the end of the “ZERO-COVID” policy. Specifically, this study explored the affective and cognitive mechanisms underlying the association between social media exposure and COVID-19 preventive behaviors. Following the abrupt transition away from ZERO-COVID policies, social media functioned not merely as a channel for information dissemination but as an important digital environment in which individuals encountered, interpreted, and responded to emerging health threats. Continuous exposure to COVID-19–related information on social media may have been associated with greater salience of health risks, thereby being associated with individuals’ cognitive risk appraisals (e.g., perceived susceptibility and severity) and affective responses such as fear. The findings showed that social media exposure is associated with increased fear of COVID-19 and risk perception. Both fear and risk perception were found to mediate the relationship between social media exposure and COVID-19 preventive behaviors. Unlike these previous studies (67, 103, 104), this study extends prior research by illustrating how individuals manage health uncertainty in digitally mediated environments when responsibility for risk assessment increasingly shifts from institutions to individuals. The results revealed that mindfulness has a significant moderation effect on the relationship between fear and preventive behaviors when the sudden end of “ZERO-COVID” policy spurred great anxiety and stress among the public. The observed associations may also reflect the distinctive social context of China’s post–ZERO-COVID transition, during which responsibility for pandemic risk management gradually shifted from institutional control toward individual decision-making. For the preceding 3 years, COVID-19 preventive behaviors were largely guided by centralized public health policies and mandatory interventions. Following the abrupt discontinuation of these restrictions, individuals faced greater uncertainty and were required to independently evaluate infection risks and determine appropriate protective actions. Within this context, social media served as the primary digital health environment where individuals encountered emerging health threats, evaluated personal risks, and constructed behavioral responses through exposure to information and social interaction. By examining social media exposure, risk perception, fear, and mindfulness during the post-ZERO-COVID transition, the findings contributed to our current understanding of psychological factors that are associated with COVID-19 preventive behaviors by moving beyond the question of whether media exposure is associated with such behaviors. Instead, it highlights how individuals navigate health uncertainty in digitally mediated environments where risk perception and behavioral decisions increasingly rely on personal information processing and self-regulation.

In accordance with the first hypothesis, social media exposure was positively associated with COVID-19 fear. Individuals who were exposed to more COVID-related content on social media were more likely to experience fear. This finding is in line with prior research that has shown social media exposure had a significant effect on negative emotions such as fear and anxiety (105). After the end of the “ZERO-COVID” policy, the death number surged at an unprecedented rate, and a significant number of Chinese people became infected within a short time. During that period, COVID-19-related content on social media was overflowing with uncertainty and risk, which may be associated with excessive fear and anxiety. The rapidity and invisibility of COVID-19 lead to a sense of unknown and lack of control, which are essential components of fear (106). Consistent with many previous findings (66, 107), fear of COVID-19 was positively associated with participants’ engagement in COVID-19 preventive behaviors. The web-based survey was completed within 1 month after the end of “ZERO-COVID” policy when many Chinese people were experiencing a higher level of fear. Fear is revealed as an important correlate of preventive behavior because preventive behaviors such as handwashing, wearing masks, and social distancing could lower one’s perceived susceptibility to COVID-19 and therefore the level of fear may decrease.

The findings indicated that risk perception mediated the relationship between exposure to COVID-19 information on social media and preventive behaviors. Social media exposure was positively associated with risk perception, which in turn, was positively associated with COVID-19 preventive behaviors. This finding suggested that a large volume of COVID-19-related content on social media, including information about the severity of the virus, misinformation, disruption, and chaos could increase individuals’ risk perception. Such social media-induced risk perception was associated with greater engagement in preventive behaviors. This finding is consistent with prior research suggesting that risk perception is important in COVID-19 preventive behaviors (108). The results also highlight the association between social media content exposure, personal risk perception, and preventive behaviors. Future studies could explore the relationship between exposure to social media platforms, such as TikTok and Weibo, and individuals’ cognitive, affective, and behavioral responses as each platform has unique features and content.

Another important finding of the present study is that mindfulness significantly moderated the association between fear and COVID-19 preventive behaviors. Specifically, the positive association between COVID-19 fear and preventive behaviors was observed among individuals with medium and high levels of mindfulness, suggesting that individual differences in self-regulatory capacity may influence how fear-related responses are translated into health-protective actions. Fear may represent an important emotional signal associated with health-protective behaviors; however, its behavioral implications may vary with individuals’ capacity to regulate threat-related emotions. When individuals have sufficient regulatory resources, fear may more likely correspond to adaptive coping responses, whereas limited regulatory capacity may be accompanied by maladaptive behaviors. From this perspective, mindfulness may serve as a self-regulatory resource that shapes how individuals respond to health threats rather than simply reducing fear itself. Previous research has suggested that mindfulness is associated with greater emotional regulation capacity and adaptive coping responses during public health crises. In the context of China’s post–Zero-COVID transition, individuals were exposed to rapidly changing health information, uncertainty, and emotionally salient content on social media. Under these conditions, mindfulness may buffer emotional reactions, allowing individuals with higher mindfulness to maintain protective behaviors even when experiencing high fear. Accordingly, individuals with higher mindfulness may be better able to associate fear-related responses with preventive behaviors. In contrast, individuals with lower mindfulness may be more likely to experience fear as an overwhelming emotional response that is less effectively translated into protective behaviors.

Theoretically, this research advances existing scholarship on COVID-19 preventive behaviors by offering novel insights into the mechanisms shaping preventive behaviors amid China’s abrupt discontinuation of the “ZERO-COVID” policy. By integrating the Extended Parallel Process Model (EPPM) and self-regulation theory, this study contributes to the literature by examining how emotional regulation may shape the relationship between threat-related responses and preventive behaviors within the EPPM framework under high-uncertainty crisis transitions and extending self-regulation theory into the domain of digital mediated environment. These findings suggest that threat appraisal alone may not fully explain behavioral responses; rather, individuals’ self-regulatory capacities may shape how threat-related emotions are associated with preventive actions. Specifically, the present study highlights the potential of mindfulness, as an intrapersonal emotional regulation strategy, to support individuals’ behavioral resilience during public health crises. This paper unpacks the underlying affective and cognitive pathways linking social media exposure to COVID-19 protective behaviors. Empirically, it systematically dissects the mediating effects of risk perception and COVID-19 fear in that focal relationship, alongside the moderating function of mindfulness in the association between COVID-19 fear and protective behavioral adherence. These findings highlight that behavioral responses to digital health risks depend not only on exposure to risk-related information but also on individuals’ capacity to regulate emotional reactions. Mindfulness may therefore represent an important psychological resource for navigating uncertainty and adapting to changing health risk environments (92).

Practically speaking, this study provides several important implications for public health practice, particularly in the context of rapidly evolving health crises. First, this study identifies mindfulness as a scalable psychological resource that bridges the gap between individuals’ health-related fear and their adaptive preventive actions. From a public health perspective, incorporating brief, accessible mindfulness-based interventions into digital health communication (e.g., short guided exercises embedded in social media platforms) may enhance behavioral compliance. During public health emergencies, such low-cost interventions can be easily scaled up to encourage public self-regulation.

Second, in post-policy transition contexts such as the abrupt relaxation of COVID-19 restrictions, information environments are often characterized by uncertainty and misinformation. Public health systems should therefore collaborate with social media platforms to implement evidence-based information curation strategies, including prioritizing authoritative content, deploying real-time myth correction, and using behavioral nudges to guide users toward reliable health information.

Third, the findings highlight the dual role of social media in relation to preventive behavior and psychological well-being. Increased exposure to COVID-19-related information on social media was associated with enhanced risk perception and more engagement in protective actions. It is also linked to fear levels, which may undermine mental well-being if left unregulated. Public health authorities should therefore move beyond “fear appeal” strategies that are often used in health and risk communication and adopt balanced communication approaches that combine risk information with clear and actionable guidance.

Finally, the results suggest that effective health communication should integrate both cognitive and emotional components. Risk perception alone is insufficient; emotional responses such as fear demonstrate a critical link to protective behaviors. However, the adaptive nature of this emotional pathway may depend heavily on individual regulatory capacities, as unregulated fear is often tied to increased maladaptive fear control. This underscores the need for communication strategies that not only inform but also support emotional coping.

The study is not without limitations. First, the data suffer from sampling bias since we used an online questionnaire, as only individuals with Internet access and a certain level of literacy can fill out the online questionnaire. The sample used in this study has a younger, educated population. The non-representative online samples could have a generalizability problem and the results of the present study may not be representative of the general population. Future studies may replicate this study using the general population or other samples such as older adults. Second, because this study used cross-sectional data, temporal ordering between social media exposure, psychological responses, and preventive behaviors cannot be established. While the proposed moderated mediation model offers important theoretical insights, establishing a causal mechanism warrants longitudinal or experimental studies. Third, social media exposure was operationalized primarily as frequency of exposure. This frequency-based measure does not capture other important dimensions of social media exposure, such as time spent on social media, content characteristics (e.g., information valence, source, and evidentiary basis), or algorithmic recommendations. Individuals with similar exposure frequencies may nevertheless experience different information environments depending on the user profile, how algorithmic systems shape users’ information environments, and usage patterns. Future research should adopt multidimensional measures of social media exposure by incorporating exposure duration, content characteristics, and digital trace data to improve the measurement validity and provide a more comprehensive assessment of individuals’ social media exposure patterns. Fourth, although efficacy beliefs are central components of the EPPM framework, the present study focused on the threat appraisal and emotional regulation processes associated with preventive behaviors. Future research should incorporate efficacy-related variables to provide a more comprehensive examination of EPPM-based mechanisms. In addition, all variables were measured using self-reported questionnaires, which may introduce common method bias and social desirability bias. Future research could combine survey data with behavioral indicators, longitudinal assessments, or digital trace data to improve measurement validity. Lastly, though this topic remains open to future follow-up research, this work conducts a valuable inquiry into how COVID-19 preventive behaviors take shape during public health policy transitions. Its findings not only extend relevant scholarship on COVID-19 preventive behaviors but also yield critical strategic guidance for frontline public health practice.

## 5 Conclusion

In conclusion, the present research highlights the role of psychological factors played in connecting social media exposure and COVID-19 preventive behaviors. It adds to the existing body of literature on the emotional, cognitive, and behavioral correlates of social media exposure. Overall, these findings underscore the complex interplay between fear, perceived risk, mindfulness, and preventive behaviors during the COVID-19 pandemic. Crucially, mindfulness may represent a psychological resource that helps individuals translate fear-related responses into adaptive behaviors. Future interventions could consider incorporating mindfulness-based strategies to help individuals manage their fear and enhance their ability to adopt recommended preventive measures.

## Data Availability

The raw data supporting the conclusions of this article will be made available by the authors, without undue reservation.
